# Differential type I interferon response and primary airway neutrophil extracellular trap release in children with acute respiratory distress syndrome

**DOI:** 10.1038/s41598-020-76122-1

**Published:** 2020-11-04

**Authors:** Jocelyn R. Grunwell, Susan T. Stephenson, Ahmad F. Mohammad, Kaitlin Jones, Carrie Mason, Cydney Opolka, Anne M. Fitzpatrick

**Affiliations:** 1 Department of Pediatrics, Emory University School of Medicine, Atlanta, GA USA; 2 Division of Critical Care Medicine, Children’s Healthcare of Atlanta at Egleston, 1405 Clifton Road NE, Atlanta, GA 30322 USA

**Keywords:** Medical research, Diseases, Infectious diseases, Respiratory tract diseases, Immunopathogenesis, Immunology, Innate immunity

## Abstract

Acute respiratory distress syndrome (ARDS) is a heterogeneous condition characterized by the recruitment of large numbers of neutrophils into the lungs. Neutrophils isolated from the blood of adults with ARDS have elevated expression of interferon (IFN) stimulated genes (ISGs) associated with decreased capacity of neutrophils to kill *Staphylococcus aureus* and worse clinical outcomes. Neutrophil extracellular traps (NETs) are elevated in adults with ARDS. Whether pediatric ARDS (PARDS) is similarly associated with altered neutrophil expression of ISGs and neutrophil extracellular trap release is not known. Tracheal aspirate fluid and cells were collected within 72 h from seventy-seven intubated children. Primary airway neutrophils were analyzed for differential ISG expression by PCR, STAT1 phosphorylation and markers of degranulation and activation by flow cytometry. Airway fluid was analyzed for the release of NETs by myeloperoxidase-DNA complexes using an ELISA. Higher STAT1 phosphorylation, markers of neutrophil degranulation, activation and NET release were found in children with versus without PARDS. Higher NETs were detected in the airways of children with ventilator-free days less than 20 days. Increased airway cell IFN signaling, neutrophil activation, and NET production is associated with PARDS. Higher levels of airway NETs are associated with fewer ventilator-free days.

## Introduction

Pediatric acute respiratory distress syndrome (PARDS) affects nearly 7% of intubated and mechanically ventilated children for acute respiratory failure^1^. While the overall mortality rate of PARDS is around 15%, the mortality rate is 35% in children with severe PARDS^1^. To date, little is known about the biological mechanisms associated with PARDS, as it remains a heterogeneous syndrome with pathology initiated by a number of diverse triggers, including pulmonary or systemic infection (sepsis), trauma, aspiration, near-drowning, and cardiac arrest^2,3^. Regardless of the trigger, neutrophils are recruited into the airways of children with PARDS where they not only combat infectious organisms and viruses, but also contribute to a hostile airway environment that may inflict further lung damage^4^. For example, in adults with acute respiratory distress syndrome (ARDS), worse clinical outcomes are associated with increased levels of both circulating and airway fluid neutrophil-related products and in dysregulated neutrophil functions^5–7^.

We have previously demonstrated that neutrophils exposed to airway fluid from mechanically ventilated children with virally-induced respiratory failure and bacterial coinfection have decreased respiratory burst and bacterial killing capacity^8^. However, the mechanisms underlying these observations are still unclear. Respiratory viruses have been shown to induce type I interferon (IFN) (IFNα/β) responses that then upregulate expression of interferon-stimulated genes (ISGs) such as *MX1*, *ISG15*, and* IFIT1*^9,10^, which influence neutrophil behavior and function^5,6^. Since viral lower respiratory tract infections are a primary trigger for PARDS, we questioned whether differential ISG expression was associated with neutrophil responses in intubated children at risk or with PARDS. We hypothesized that children with PARDS would have a greater type I IFN response resulting in more neutrophil extracellular trap (NET) release.

## Materials and methods

This prospective, observational study was performed in the thirty-six-bed academic medical/surgical Pediatric Intensive Care Unit (PICU) at Emory University/Children’s Healthcare of Atlanta at Egleston from January 2018 through February 2020. The study was approved by the Institutional Review Board at Emory University (IRB 00034236 and IRB 00113035) and all methods were carried out in accordance with relevant guidelines and regulations in the Declaration of Helsinki. Informed consent was obtained from the parents of all subjects prior to collection and use of their samples.

### Study population

All patients admitted to the PICU who were greater than 2 days, with a corrected gestational age of at least 40 weeks, and were younger than 18 years old that met criteria for being at risk or having PARDS, as defined by the Pediatric Acute Lung Injury Consensus Conference (PALICC), were screened for eligibility^11^. Children had to have lung injury within 7 days of a known clinical insult, new infiltrate(s) consistent with acute pulmonary parenchymal disease on chest imaging and be receiving oxygen delivered either non-invasively or invasively to maintain an oxygen saturation between 88 and 97%. Children were excluded if they had any peri-natal related lung disease, respiratory failure fully explained by cardiac failure or fluid overload, chronic respiratory failure with mechanical ventilation via a tracheostomy or RAM cannula, confirmed immunodeficiency disorder, immunosuppression from chemotherapy for an oncologic process, chronic immunosuppression in a bone marrow transplant or solid organ transplant recipient, no parent or legal guardian present to provide written informed consent, or the attending physician did not wish the patient to participate in the study. Children were enrolled as controls if they were endotracheally intubated for airway protection and without lung pathology or signs of systemic infection or inflammation. For example, children selected as controls were electively intubated to facilitate radiologic imaging, for non-airway or cardiothoracic surgeries, and for airway protection following an acute ingestion or altered mental status without suspicion for infection or systemic inflammation.

### Data collection

Clinical data were abstracted from the medical record onto a standardized form. Variables included demographics; fraction inspired oxygen, mean airway pressure, arterial oxygen saturation or arterial oxygen pressure used to calculate an oxygen saturation index (OSI) or oxygenation index (OI), respectively; laboratory and microbiology results; length of mechanical ventilation and need for reintubation; length of PICU stay, use of high frequency oscillatory ventilation (HFOV) or extracorporeal life support (ECLS), and vital status. Severity of illness was determined by the Pediatric Risk of Mortality (PRISM)-III and pediatric Logistic Organ Dysfunction (PELOD) scores were calculated within 24 h of intubation^12,13^. Need for mechanical ventilation to 28-days was monitored to calculate ventilator-free days^14^. Lung injury severity was categorized according to PALICC criteria^11^.

### Tracheal aspirate collection

Tracheal aspirates were obtained from patients on conventional mechanical ventilation by instilling 1–5 mL of sterile saline through the inline Ballard suction catheter and into a sterile Luken’s trap as part of routine suctioning per published protocols^8^. Children who were mechanically ventilated with HFOV were suctioned only if clinically indicated and approved by the attending physician. Tracheal aspirate samples were immediately placed on ice for transport to the laboratory for processing.

### Airway sample processing

Tracheal aspirate was gently dissociated using repeated passage through an 18G needle after the addition of 6 ml of PBS-EDTA. Dissociated tracheal aspirate was then centrifuged at 800×*g* to generate a cell pellet and a fluid fraction. The fluid fraction was spun at 3000×*g* to generate cell-free airway supernatant (ASN), aliquoted, and stored at − 80 °C^8^. Airway cells were resuspended in PBS-EDTA and cell density was quantified using a Countess hemocytometer using trypan blue exclusion to determine cell viability. Cell purity was also assessed by cytospin preparations and Diff-Quik staining of airway cells. Cell viability was also assessed using a Live/Dead Aqua stain in the surface staining flow cytometry panel.

### Gene expression assays

RNA was isolated from airway cells stored at − 80  C in RNA Later using the NucleoSpin RNA II kit with on-column genomic DNA digestion according to the manufacturer’s protocol (Takara, Mountain View, CA). RNA was quantified using a NanoDrop Fluorospectrometer (Therma Scientific). RNA integrity (RIN) was measured at the Emory Integrated Genomics Core on an Agilent 2100 bioanalyzer (see Supplementary Table S1 online for RIN values). Only samples with a RIN value greater than 7 that passed quality control were analyzed. cDNA was synthesized using a High Capacity cDNA Reverse Strand Synthesis kit (Applied Biosystems, Foster City, CA). Specific genes were preamplified by PCR using the cDNA with TaqMan PreAmp Master Mix and commercially available TaqMan Assays (Applied Biosystems ID; UniGene ID, IFIT: Hs03027069_s1; Hs.20315, ISG15: Hs01921425_s1; Hs.458485, MX1: Hs00895608_m1; Hs.517307) according to the manufacturer’s protocol (Applied Biosystems/Thermo Fisher). Quantitation PCR was performed using TaqMan Gene Expression assays and Master Mix on a StepOnePlus Real Time PCR System (Applied Biosystems/Thermo Fisher). The human housekeeping gene B2M (Hs99999907_m1; Hs.534255) was used for normalization. Each measurement was performed in duplicate and averaged. The 2^(−ΔΔCt)^ method was used to calculate the relative differences in gene expression. The data are reported as the mean ± SEM of individual patient’s airway cells.

### Cell staining and flow cytometry

Airway cells collected from human subjects were preincubated with human TruStain FcX receptor blocking solution (Biolegend, San Diego, CA) and Live/Dead Aqua (Thermo Fisher) for 10 min on ice in the dark followed by surface staining with CD63-Pacific Blue (Clone: H5C6, BioLegend), CD41a-BV510 (Clone: HIP8, BD Biosciences), S1PR3-AF488 (Clone: 776808, R&D Systems), CD66b-PerCPCy5.5 (Clone: G10F5, BioLegend), Arg1-PE (Clone: 14D2C43, BioLegend), and CD16-APCCy7 (Clone: 3G8, BioLegend) for 30 min on ice in the dark. Samples were treated with BD PhosFlow Lyse/Fix Buffer (BD Biosciences, San Jose, CA), washed twice with PBS-EDTA, and stored at 4 °C in the dark until acquisition on a CytoFLEX flow cytometer (Beckman Coulter, Indianapolis, IN)^8^. Compensation using AbC Total Antibody and ArC Amine-Reactive Compensation Beads (Thermo Fisher Scientific), gating and analysis were performed with FlowJo v.10 (Tree Star, Ashland, OR)^8^. Single cells were separated from doublet cells by gating on forward scatter area versus side scatter area profiles^8^. Live cells were then selected by exclusion of dead events positive for the dye Live/Dead Aqua, and platelets or platelet/leukocyte aggregates were excluded by staining for CD41a^+^ events^8^. CD66b^+^ neutrophils were then confirmed by gating on CD66b versus side scatter area^8^. Mean fluorescence intensities of surface markers are reported for CD66b^+^ neutrophils. To determine the background mean fluorescence intensity signal in the reporter channels (PB450, AF488, PE), we report background mean fluorescent intensities in CD66b^+^ neutrophils using fluorescent minus controls that were only stained for CD41a, Live/Dead Aqua, CD66b, and CD16 (Supplementary Figure S1 online)^8^. The median (IQR) for the fluorescent-minus reporter channels are for PB: 562 (3847–13,315, *n* = 21), AF488: 3949 (2586–11,400, *n* = 21), and for PE: 378 (207–993, *n* = 20) and are below the 10th percentile value of the No PARDS and Yes PARDS data. Due to limited numbers of cells in some tracheal aspirate samples, not every sample had a fluorescence minus control performed. For some samples, airway cells were fixed, permeabilized, and stained intracellularly with STAT1-PE (Clone: D1K9Y) and Phosphorylated (Tyr701)-STAT1-AF488 (Clone:58D6, Cell Signaling Technologies, Danvers, MA) and then analyzed with by flow cytometry (FACSCalibur, BD Biosciences, Franklin Lakes, NJ). Expression analysis was performed using FlowJo version 10 software (FlowJo, LLC, Ashland, OR).

### Quantification of NETs

To quantify NETs from the airway fluid, a capture ELISA myeloperoxidase (MPO) associated with DNA was performed as previously described^15^. For the capture antibody, Human MPO ELISA kit (R&D Systems, Minneapolis, MN) was used according to the manufacturer’s instructions. We added 200 µL of airway fluid to the wells and incubated for 1 h. After washing 3 times with 300 µL of wash buffer, 100 µL incubation buffer containing a peroxidase-labeled anti-DNA mAb (component 2, Cell Death ELISA^PLUS^, Roche) was used and expressed as relative fluorescence units (RFU). Each sample’s RFU value was normalized to the mean and standard deviation (SD) for each experiment using the standardization formula: (1 + (sample value − mean experiment value)/SD experiment value) for each experiment.

### Statistical analysis

Statistical analyses were performed using JMP Pro 14 (SAS Institute, Cary, NC) and GraphPad Prism 8 for Windows. Unless otherwise stated, comparisons between samples of children with versus without PARDS were made using a two-tailed Mann–Whitney U test for nonparametric data. For PCR studies, a ROUT outlier test using a 1% false discovery rate was performed prior to a two-tailed Mann–Whitney U test. Statistical significance was defined as a *p *value less than 0.05.

## Results

### Subject characteristics

Seventy-seven patients were enrolled into the study within 72 h of intubation and mechanical ventilation. Of these 77 children, 42 (54.6%) had PARDS and 35 (45.4%) did not have PARDS. Table 1 shows the demographics and clinical characteristics of the 77 enrolled patients. The respiratory viral PCR panel and respiratory culture results from the clinical microbiology lab are reported in Supplementary Table S2 online. Children with PARDS were more likely to be supported by extracorporeal life support (ECLS), had a longer duration of mechanical ventilation, and spent more days in the hospital and the PICU (Table 1). There were no significant differences in severity of illness (PRISM III) or organ dysfunction (PELOD) scores, the absolute number or percentage of alive neutrophils from children based on PARDS status (Table 1).

**Table 1:** Demographic and clinical characteristics of study participants.

Characteristic	PARDS status		*p *value
	No, *n* = 35 (45.4%)	Yes, *n* = 42 (54.6%)	
Age (years), median (IQR)	0.53 (0.12–2.32)	1.07 (0.47–2.19)	0.0904
**Sex, n (%)**			
Female/male	14 (40.0)/21 (60.0)	18 (42.9)/24 (57.1)	0.8000
**Race/ethnicity, n (%)**			
Black, non-Hispanic	20 (57.1)	18 (42.8)	0.1089
White, non-Hispanic	13 (37.1)	17 (40.5)	
Multiple, non-Hispanic	0 (0)	5 (11.9)	
Declined, non-Hispanic	0 (0)	1 (2.4)	
Declined, Hispanic	1 (3.7)	0 (0)	
Unknown, Hispanic	1 (3.7)	0 (0)	
**Severity of lung injury**^**a**^**, *n (%)***			
Mild	NA	16 (38.1)	
Moderate	NA	13 (30.9)	
Severe	NA	13 (30.9)	
**Severity of illness scores, median (range)**			
PRISM III	12 (8–17)	16 (11.75–20)	0.0579
PELOD	6 (4–7)	6 (5–9)	0.0735
Ventilator days, median (IQR^d^)	3 (2–6)	7 (4–12.25)	< 0.0001
Extracorporeal life support, *n* (%)	0 (0)	7^c^ (16.7)	0.0026
**Length of stay, median (IQR)**			
PICU (days)	5 (3–9)	0 (6–14)	< 0.0001
Hospital (days)	10 (5–12)	14.5 (8.75–22)	0.0024
**28-day mortality, n (%)**			
Dead	0 (0)	3 (7.14%)	
**Viral respiratory panel, n (%)**			
Positive	26 (74.3)	31 (73.8)	
No virus detected	1 (2.8)	6 (14.29)	
Not assessed	8 (22.8)	4 (9.5)	
Multiple viruses	4 (11.4)	4 (9.5)	
**Respiratory culture, n (%)**			
No growth	5 (14.3)	8 (19.0)	
Bacterial growth	19 (54.3)	28 (66.7)	
Yeast growth	0 (0)	1 (2.4)	
Not assessed	11 (31.4)	6 (14.3)	
Virus + bacterial coinfection	18 (51.4)	21 (50.0)	
**Airway neutrophils, median (IQR)**			
Neutrophil count (× 10^6^)	*n* = 24 6.5 (2.4–20.3)	*n* = 19 4.3 (0.9–7.8)	0.1039
Neutrophils (%)	*n* = 25 87.2 (79.2–93)	*n* = 19 87.0 (69.2 – 95.8)	0.7223

^a^Severity of lung injury is defined using oxygenation index or oxygen saturation index using the Pediatric Acute Lung Injury Consensus Conference definitions (ref).

^b^NA, not applicable.

^c^One child classified as Moderate PARDS on Day 1 was cannulated to ECLS; the remaining 6 children were classified as Severe PARDS on Day 1.

^d^IQR, 25th–75th interquartile range. Comparisons were made with a two-tailed Mann–Whitney U Test for continuous variables or a Chi-square test for categorical variables.

### Activation status of airway cells

**Figure 1:**
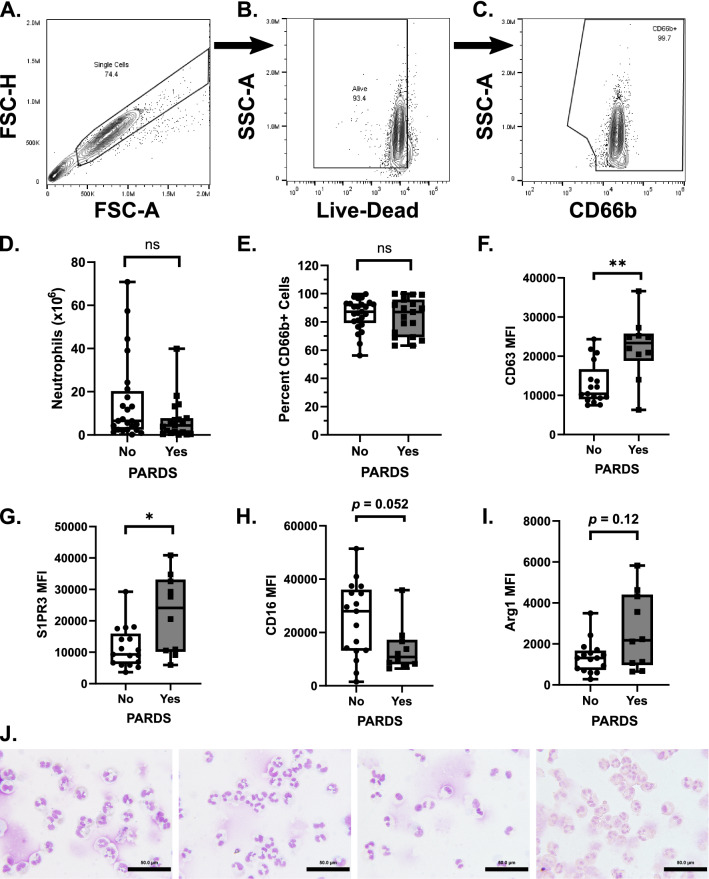
Neutrophil activation and degranulation markers in the tracheal aspirate samples from children with acute respiratory failure with and without PARDS. The gating strategy for single, living, CD66b^+^ neutrophils is shown (**A–C**). The total number of neutrophils in the sample (**D**) was determined by multiplying the total number of cells in the tracheal aspirate sample by the percentage of living CD66b^+^ cells (neutrophils) in the sample (**E**). The mean fluorescence intensity (MFI) of (**F**) CD63 (*n* = 17, No PARDS, *n* = 10, Yes PARDS), (**G**) sphingosine 1-phosphate receptor 3 (S1PR3) (*n* = 17, No PARDS, *n* = 10, Yes PARDS), (**H**) CD16 (*n* = 17, No PARDS, *n* = 10, Yes PARDS), and (**I**) Arginase 1 (Arg1, *n* = 16, No PARDS, *n* = 10, Yes PARDS) on the surface of airway neutrophils of children with PARDS (gray boxplots) compared with children without PARDS (white boxplots) are shown. Each circle (No PARDS) and each square (Yes PARDS) represent an individual patient. A cytospin and Diff-Quik stained processed airway sample is shown (**J**). Scale bars indicate 50 microns. Two-tailed Mann–Whitney U test. **p < 0.01, *p < 0.05.

This study was a survey of the activation status of neutrophils is PARDS. We assessed markers of airway neutrophil activation by flow cytometry. The gating strategy for selecting airway neutrophils is shown (Fig. 1A–C). There was no difference in total number (Fig. 1D) or percent CD66b^+^ neutrophils (Fig. 1E) in intubated children with or without PARDS. Children with PARDS also had increased neutrophil surface expression of CD63, a marker of primary granule exocytosis (Fig. 1F), and sphingosine 1-phosphate receptor 3 (S1PR3), a protein that forms a heterodimer with the neutrophil IL8-induced chemotaxis receptor, CXCL1, and is detected with increased abundance in blood neutrophils from adults with bacterial pneumonia (Fig. 1G)^16^. There was no significant difference in surface expression of the type III Fcγ receptor, CD16 or arginase I (Arg1), an enzyme stored in the primary and tertiary granules of human neutrophils (Fig. 1H–I). Examples of cytospin preparations stained with Diff-Quik from four unique patient tracheal aspirates show the predominance of neutrophils in the airway samples (Fig. 1J).

**Figure 2:**
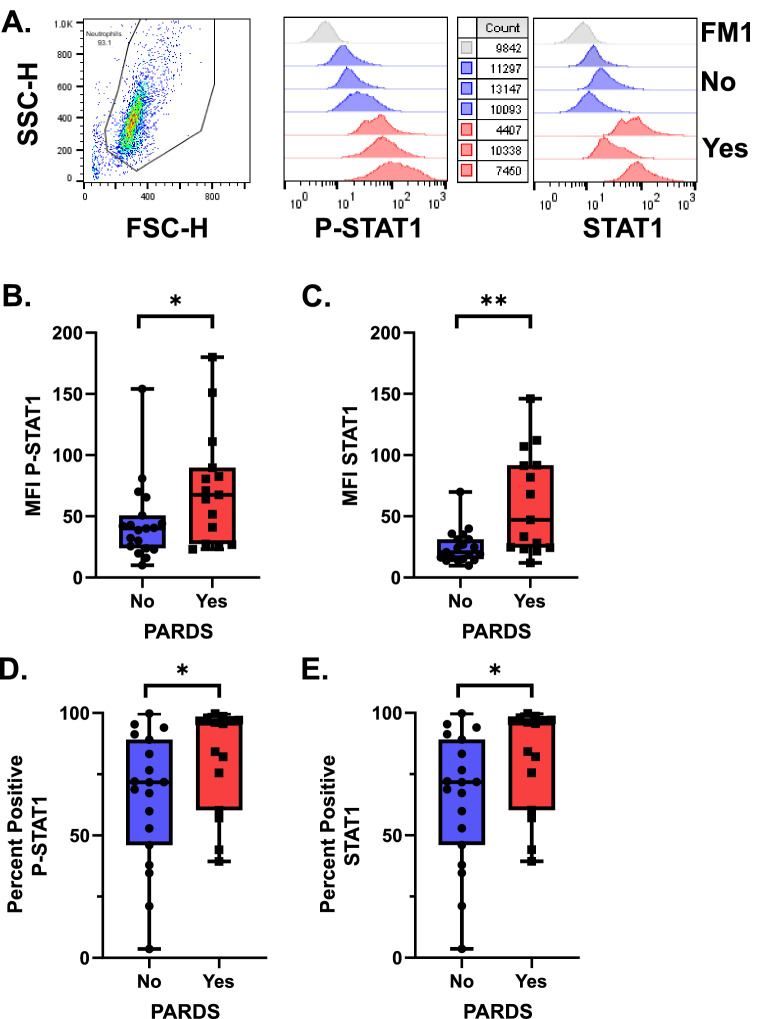
Phosphorylated STAT-1 (P-STAT1 (Y701)) and STAT1 expression in primary airway cells in children with and without PARDS. Cells were gated on forward and side-scatter. Histograms of primary flow data along with cell counts are noted. Histogram data is scaled using the modal function in FlowJo analysis software (**A**). Boxplots of the mean fluorescence intensity (MFI) of P-STAT1 (**B**) and STAT-1 (**C**) along with the percent cells positive for P-STAT1 (**D**) or STAT1 (**E**) are shown. The fluorescence minus 1 (FM1) histogram was used to draw the percent positive gate for P-STAT1 and STAT-1, respectively. FM1 (gray histogram), No PARDS (circles/blue boxplot and blue histograms) and PARDS (squares/red boxplot) and red histograms). For all analyses, *n* = 19 (No PARDS), *n* = 15 (Yes PARDS). Two-tailed Mann–Whitney U test. *p < 0.05.

**Figure 3:**
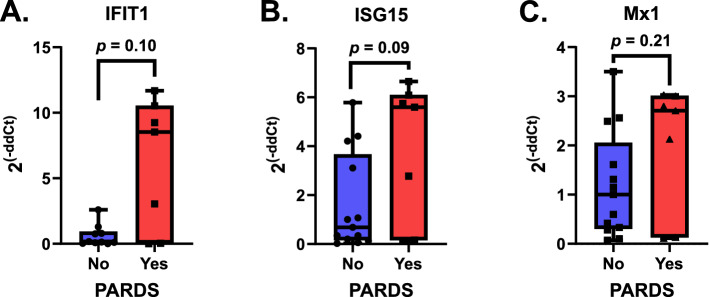
Anti-viral and interferon (IFN) stimulated gene (ISG) expression in control children and in children with and without PARDS. (**A**) IFIT1 (IFN Induced proteins with Tetraicopaptide repeats), *n* = 10 with 3 outliers removed (No PARDS), *n* = 7 (Yes PARDS), (**B**) ISG15, *n* = 12 with 1 outlier removed (No PARDS), *n* = 7 (Yes PARDS), (**C**) MX1, *n* = 13 with no outliers (No PARDS), *n* = 7 (Yes PARDS). Two-tailed Mann–Whitney U test. The *p* values are noted above the boxplots.

We next determined whether the type I interferon (IFNα/β) signaling pathway was activated by measuring the phosphorylation of signal transducer and activator of transcription 1 (STAT1) by intracellular staining of fixed airway samples. Airway cells from children with PARDS had increased expression of phosphorylated-Y701 STAT1 (P-STAT1) and STAT1 compared with children who did not meet PARDS criteria (Fig. 2). Cells were gated on forward and side scatter and representative histograms for both P-STAT1 and STAT1 are shown (Fig. 2A). Quantification of both the mean fluorescence intensity (MFI) and percent positive cells are summarized in Fig. 2B–E. Children with PARDS have higher levels of STAT1 and P-STAT1 compared with children without PARDS. Activation of the type I interferon signaling pathway results in increased expression of many IFN-stimulated genes (ISGs)^17^. Three ISGs, IFIT1, ISG15, and Mx1, were used to identify activation the type I IFN signaling pathway^5,6^. ISG expression of children with versus without PARDS were compared. ISG expression was variable; however, children with PARDS did not show a significant difference in expression level of ISG15, IFIT1 or Mx1 compared to children without PARDS (Fig. 3). Our results show that markers of neutrophil degranulation and activation are elevated in children with versus without PARDS. In addition, activation of the type I IFN signaling pathway, as indicated by increases in P-STAT1, and ISG15 expression occurs in some children with PARDS.

### Airway NET levels and clinical outcomes

**Figure 4:**
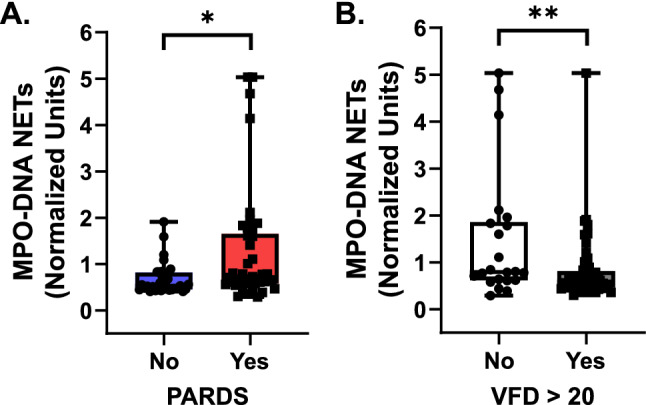
Airway neutrophil extracellular trap (NET) release by PARDS status and ventilator-free days. NET release as measured by MPO-DNA enzyme-linked immunosorbent assay (**A**) from cell-free tracheal aspirate samples of children without PARDS (blue boxplot, *n* = 33, where each circle is an individual patient) versus with PARDS (red boxplot, *n* = 42, where each square is an individual patient). (**B**) MPO-DNA complexes in tracheal aspirates were stratified by those children who did not have more than 20 ventilator-free days (VFD) (white boxplot, *n* = 22, where each circle is an individual patient) and those children who did have more than 20 VFD (gray boxplot, *n* = 53, where each square is an individual patient). Boxplots depict median (line), 25th–75th interquartile range (box edges), and min to max values (whiskers). Values are normalized to a standardized value of 1 and analyzed using a two-tailed Mann–Whitney U test, *p < 0.05, **p < 0.01.

Airway NET release was quantified in airway fluid by (MPO)-DNA complexes^18^. Relative units of NETs were significantly higher in the airway fluid of children with versus without PARDS (Fig. 4A). Higher airway NET levels were associated with fewer ventilator-free days (F = 21.20, *p *value < 0.0001), a lower proportion of children with ventilator-free days over 20 days (Fig. 4B), and a longer PICU length of stay (F = 19.25, *p *value < 0.0001). Children with PARDS had fewer ventilator-free days (median: 21 days, IQR: 14–24 days) compared with children without PARDS (median: 25 days, IQR: 22–26 days, *p* < 0.0001). The proportion of children with PARDS had fewer ventilator-free days over 20 days compared with children without PARDS (33/35, 94.3% vs. 22/42, 52.4%, Chi-squared likelihood ratio test = 18.67, *p *value < 0.0001).

## Discussion

**Figure 5:**
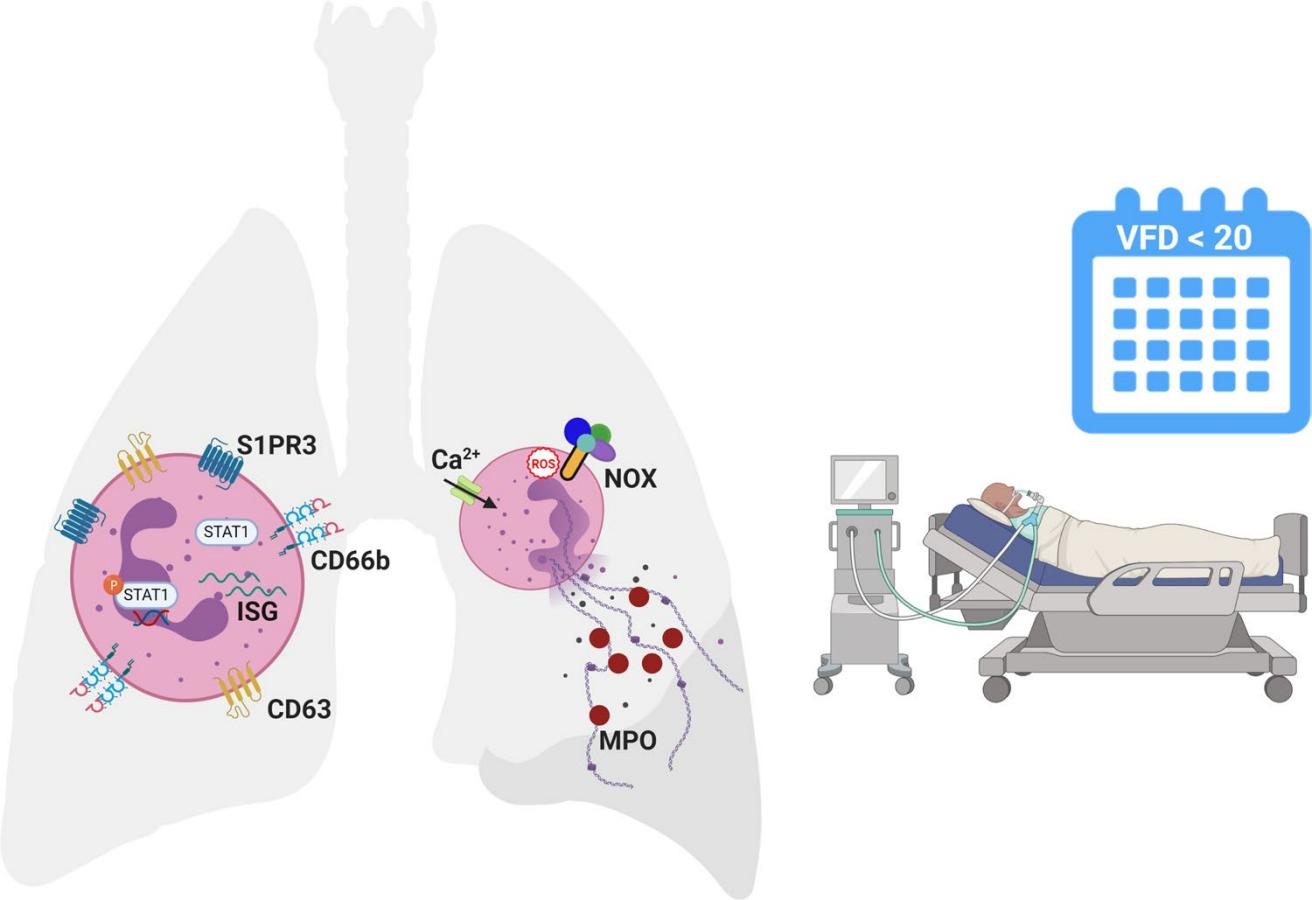
Airway neutrophils are activated in intubated children with compared to children without PARDS. Surface expression of the primary granule exocytosis marker, CD63, and the lipid signaling G protein-coupled receptor, sphingosine-1-phosphate receptor 3 (S1PR3), are higher in children with versus without PARDS. The type I interferon (IFN) signaling pathway transcription factor, STAT1, is upregulated and phosphorylated, and transcript levels of ISG15 is increased in children with versus without PARDS. Neutrophil extracellular trap (NET) release is regulated by a NADPH oxidase (NOX) respiratory burst (reactive oxygen species (ROS) triggered mechanism and an intracellular calcium-dependent trigger. It is not known which trigger dominates in the airways of children with PARDS. Children with PARDS have elevated levels of NETs in their airways as detected by myeloperoxidase (MPO)-DNA complexes in our study. Elevated NET levels are associated with a higher number of ventilator-free days (VFD) over 20 days in a 28-day period (i.e. if the child survived, then they were more likely to spend ≥ 7 days endotracheally intubated and mechanically ventilated). Created with BioRender.com using the web version, which may be accessed at https://biorender.com/, with a paid individual subscription granting permission to publish in journals.

Acute LRTI are the trigger for the majority of PARDS^1^. Viral and bacterial infections activate type I IFN signaling pathways resulting in an increase in antiviral ISG expression and proinflammatory responses critical for host defense^10,19^. A finely tuned type I IFN response is crucial to a host as an excessive or prolonged type I IFN response may lead to impaired gas exchange in the lung due to cellular and tissue destruction^20^. IFNs prime mature neutrophils for NET release upon stimulation with a second stimulatory signal^21^. Although antimicrobial proteins expressed on NETs can inactivate pathogens and prevent viral spread to neighboring cells, the lung is vulnerable to the damage that histones and proteolytic enzymes contained within NETs can inflict to host tissue. Therefore, excessive and prolonged type I IFN signaling may contribute to the production of NETs which fill the alveolar spaces, lead to increased inflammation, and result in impaired lung function, which are the hallmarks of PARDS. Our study assessed neutrophil activation, the differential expression of ISGs and activation of the STAT1 signaling pathway, and NETosis in the airways of intubated children with acute respiratory failure due to lower respiratory tract infections. We found increased neutrophil degranulation markers, phosphorylation of STAT1 (Y701), and NETs, as measured by MPO-DNA complexes, in the airways of children with PARDS compared with children without PARDS. Higher levels of airway NETs were associated with fewer ventilator-free days. Our findings are summarized in Fig. 5.

The timing and magnitude of the type I IFN response are important in modulating the immune response to viral infection^22,23^. At higher levels, ISGs contribute to dysregulated lung inflammation, disease progression, and are linked to worse clinical outcomes^20^. For example, in a mouse model of SARS-CoV infection, the delayed expression of high levels of type I IFN in the presence of high viral titers resulted in lethal pneumonia^22^; however, early treatment with type I IFN within the first six hours of SARS-CoV infection was protective. In Influenza A virus, disease severity and progression are associated with overshooting the IFN-driven inflammatory response whereby exogenous supplementation with type I IFN correlated with increased morbidity and mortality^24,25^. RSV also induces high levels of pro-inflammatory cytokines directly related to type I IFN, and mice that lack IFNAR have less proinflammatory cytokine release resulting in a less severe disease course following RSV infection^26^. By contrast, there was no effect of IFNAR deletion on pathogenicity of mice infected with SARS-CoV; however, STAT1 deficient mice showed increased susceptibility, prolonged viral shedding and mortality^27^.

Differential type I IFN and ISG expression are associated with neutrophil dysfunction and worse clinical outcomes^5,6^. For example, high expression of a panel of ISGs (*MX1*, *IFIT1*, *ISG15*) from circulating neutrophils from a subgroup of adults with ARDS compared with normal ISG expressing neutrophils had reduced migration toward the neutrophil chemokine interleukin-8 (IL-8), decreased p38 MAP kinase phosphorylation, superoxide anion release, IL-8 release, and a shift from necrotic to apoptotic cell death that was associated with a diminished capacity to kill *Staphylococcus aureus*, but not *Pseudomonas*^5^. Subsequent hierarchal clustering analysis of the aforementioned ISG panel from circulating neutrophils of ARDS patients within 72 h of initiation of mechanical ventilation showed that both High- and Low-range expression had fewer 28-day ventilator-free and ICU-free days and higher 90-day mortality compared with Mid-range ISG expressing patients^6^. These data suggest that a targeted middle ground for IFN levels exists to benefit the host. An IFN signal below a lower threshold would result in an ineffective antiviral host response, while an IFN signal above an upper threshold would trigger a detrimental inflammatory host response. IFN signaling outside the target zone would increase lung inflammation and ARDS severity^5,6,20^. By contrast, our findings are from airway cells with a neutrophil predominance rather than from circulating neutrophils for adults with ARDS^5,6^. Additionally, we assessed each ISG, IFIT1, ISG15, and Mx1, individually rather than in aggregate, and we note that ISG expression is highly variable in children with versus without PARDS. Finally, the release of NETs was not assessed in the aforementioned studies.

Excessive NET formation has been reported in the serum and bronchoalveolar lavage fluid from adults with ARDS; however conclusions with respect to clinically relevant outcomes are difficult to draw due to the heterogeneity in study design^18,28,29^. NETs play prominent roles in bacterial pneumonia^30–32^, RSV bronchiolitis^33,34^, influenza pneumonia^31,35,36^, sepsis^37–40^, SARS-CoV2^41^, small-vessel vasculitis^15^, systemic lupus erythematosus^42,43^, and transfusion-related acute lung injury^44^. NETs induce dose-dependent cytotoxic effects on human alveolar epithelial cells due to histone and myeloperoxidase induced damage to alveolar epithelial and endothelial cells^45,46^. NETs also influence the macrophage function^47^, T cell proliferation^48^, and amplify IFN production by plasmacytoid dendritic cells^42^. Conversely, interferons influence the process of NETosis in mature neutrophils^21,49^.

High IFN/ISG expression polarizes neutrophils to an activated “N1” phenotype that can lead to increased NET production, degranulation, and influence neutrophil interactions with other immune and airway epithelial cells^50^. Interferonopathies, autoimmune diseases, and tumor-associated neutrophils are all examples where high IFN/ISG levels influence neutrophil activation and function^42,51^. Priming of neutrophils with IFN-α and subsequent stimulation with C5a resulted in increased STAT1 phosphorylation at tyrosine 701 and NET production in mature neutrophils^21^. Activation of neutrophils by type I IFNs led to increased NETosis that triggered biofilm formation by *Pseudomonas aeruginosa* and persistence in the lung^50^. NET production, like IFN signaling, exists in a balance. Excessive NET release can lead to the systemic spread of inflammation through platelet interactions and result in multiple organ dysfunction^37,39,52^; however, depletion or defective NET production can lead to the spread of infection early in the course of disease^40^.

Several mechanisms regulating the formation of NETs are known and include NADPH oxidase (NOX2) dependent and calcium channel NOX-independent NETosis^53–56^. NETosis is driven by NET-specific kinases that regulate transcription initiation in neutrophils. For example, ERK was shown to differentially regulate NOX-dependent NETosis^54^. Interestingly, Khan and colleagues also noted that STAT1 was a transcription factor that was upregulated in the NOX-dependent NETosis pathway^54^. We were not able to study the relative importance of NOX-dependent versus calcium influx (NOX-independent) mechanisms on NET formation given that NETosis has already occurred by the time of tracheal aspirate sampling in our patients. Additionally, we did not quantify the relative amounts of neutrophils undergoing NETosis versus apoptosis. Quantifying neutrophil apoptosis should be part of future studies as NOX-dependent NETosis is dependent on the activation of the kinase Akt to suppress apoptosis and switch to NETosis^57^.

While we detected higher type I IFN signaling and NET production in the airways of children with versus without PARDS, due to the clinical nature of our study, we are not able to attribute causation of neutrophil dysfunction to high ISG expression in study participants. There are several additional limitations to our study. First, this is a single-center study with a limited sample size; however, despite the limited number of children studied, we were able to detect differences in neutrophil type I IFN signaling pathway phosphorylation and function in intubated children with versus without PARDS. The majority of children in this study were at risk for developing PARDS, with the remaining children being equally distributed amongst mild, moderate and severe PARDS categories; however, the degree of PARDS severity was not associated with neutrophil dysfunction due to heterogeneity in neutrophil function and a limited sample size. We also excluded immunocompromised patients from our study which is a major risk factor associated with PARDS-related mortality^58–60^. Second, there is heterogeneity in the identity of viral and bacterial pathogens infecting study participants precluding any conclusions regarding the influence of organism on study results. We did not stratify the patients based on bacterial growth in an endotracheal respiratory culture as there is controversy regarding whether this is a true bacterial coinfection versus merely codetection of bacterial and viral organisms^61^. Thirdly, we are not powered to detect differences in mortality associated with higher ISG gene expression as seen in the adult ARDS cohort; however, we did detect a significant difference in ventilator-free days in children with a higher airway NET burden^6^. We are limited in the ability to study the mechanisms regulating airway NET release as NETosis has already occurred in patients at the time of sample collection’ however, network analysis of transcription factor signaling pathways from recovered airway neutrophils could be performed as previously described^54^. Additionally, future experiments using blood-derived neutrophils from healthy donors could be incubated in patient airway fluid to simulate the airway environment with PMA and calcium-ionophore stimulation, and appropriate NOX inhibition, to study mechanistic regulation of NET formation. Finally, we did not explore the role of other signaling pathways, such as that initiated through the sphingolipid receptor or other kinase signaling cascades, as mechanisms involved in the pathogenesis of NETs or PARDS severity.

In summary, we describe the differential type I IFN signaling based on the presence or absence of PARDS and show that airway levels of NETs are higher in children with versus without PARDS. Higher levels of airway NETs are associated with fewer ventilator-free days. Future work will explore the influence of type I IFN signaling, NETs and the PARDS airway environment on macrophage and T-cell activation and function. Defining underlying mechanistic differences in children with acute respiratory failure due to lower respiratory tract infections is needed to move beyond supportive care and toward targeted personalized therapies for pediatric patients with moderate/severe PARDS.

## Supplementary information

Supplementary Information.

## Data Availability

The datasets generated during and/or analyzed during the current study are available from the corresponding author on reasonable request.

## Abbreviations

IFN: Interferon
MPO: Myeloperoxidase
NETs: Neutrophil extracellular traps
PARDS: Pediatric acute respiratory distress syndrome
PICU: Pediatric intensive care unit
