# Supplementary material for: Differential type I interferon response and primary airway neutrophil extracellular trap release in children with acute respiratory distress syndrome
Source: Sci Rep. 2020 Nov 4;10:19049. doi: 10.1038/s41598-020-76122-1 (PMC7642368; doi:10.1038/s41598-020-76122-1)
Supplement: Supplementary file 1 — Supplementary Information. [file 41598_2020_76122_MOESM1_ESM.docx]

**Differential Type I Interferon Response and Primary Airway Neutrophil Extracellular Trap Release in Children with Acute Respiratory Distress Syndrome**

**ONLINE DATA SUPPLEMENT**

Jocelyn R. Grunwell, Susan T. Stephenson, Ahmad F. Mohammad, Kaitlin Jones, Carrie Mason, Cydney Opolka, and Anne M. Fitzpatrick

**Supplementary Table S1. Tracheal Aspirate Cell Sample RNA Integrity Number (RIN)**

**Supplementary Table S2. Respiratory Viral Panel and Culture Results**

**Supplementary Figure S1. Comparison of negative antibody staining controls and patient airway samples.**

**Supplementary Table S1. Tracheal Aspirate Cell Sample RNA Integrity Number (RIN)**

| **Sample ID** | **PARDS Status** | **RIN** |
| --- | --- | --- |
| 21 | No | 7.9 |
| 22 | No | 8.6 |
| 23 | No | 9.2 |
| 27 | No | NA |
| 34 | No | 8.7 |
| 33 | No | 8.4 |
| 36 | No | 2.1 |
| 37 | No | 7.5 |
| 38 | No | 9.7 |
| 44 | No | 7.0 |
| 48 | No | 7.8 |
| 52 | No | 5.1 |
| 53 | No | 3.2 |
| 25 | Yes | 8.8 |
| 29 | Yes | 8.8 |
| 32 | Yes | 8.9 |
| 42 | Yes | 8.3 |
| 45 | Yes | 9.1 |
| 46 | Yes | NA |
| 49 | Yes | 8.8 |
| 51 | Yes | NA |

NA = not available due to lack of sample

**Supplementary Table S2. Respiratory Viral Panel and Culture Results**

|  | **PARDS Status** | |  |
| --- | --- | --- | --- |
| **Characteristic** | **No** | **Yes** | **Total** |
| **Viral Panel, *n* (% of column total)** |  |  |  |
| Adenovirus | 0 | 2 (5.6%) | 2 (3.0%) |
| Coronavirus NL63 | 0 | 1 (2.8%) | 1 (1.5%) |
| Coronavirus HKU1 | 1 (3.3%) | 1 (2.8%) | 2 (3.0%) |
| Human metapneumovirus (HMPV) | 0 | 2 (5.6%) | 2 (3.0%) |
| Influenza A | 5 (16.7%) | 6 (16.7%) | 11 (16.7%) |
| Influenza B | 2 (6.7%) | 1 (2.8%) | 3 (4.5%) |
| *Mycoplasma pneumoniae*^b^ | 0 | 1 (2.8%) | 1 (1.5%) |
| Parainfluenza 1 | 0 | 1 (2.8%) | 1 (1.5%) |
| Parainfluenza 3 | 0 | 1 (2.8%) | 1 (1.5%) |
| Rhinovirus/Enterovirus | 13 (43.3%) | 7 (19.4%) | 20 (30.3%) |
| Respiratory syncytial virus (RSV) | 9 (30.0%) | 13 (36.1%) | 22 (33.3%) |
| **Sum Positive Viral Panel, *n* (% row total)** | **30 (45.5%)** | **36 (54.5%)** | **66 (100%)** |
| **Respiratory Culture, *n* (% of column total)** |  |  |  |
| *Enterococcus faecalis* | 0 | 1 (2.8%) | 1 (1.6%) |
| *Haemophilus influenzae* | 9 (36.0%) | 6 (16.7%) | 15 (24.6%) |
| *Klebsiella pneumoniae* | 0 | 1 (2.8%) | 1 (1.6%) |
| *Moraxella catarrhalis* | 5 (20.0%) | 3 (8.3%) | 8 (13.1%) |
| *Methicillin-resistant Staphylococcus aureus* | 2 (8.0%) | 5 (13.9%) | 7 (11.5%) |
| *Methicillin-sensitive Staphylococcus aureus* | 4 (16.0%) | 6 (16.7%) | 10 (16.4%) |
| *Streptococcus pneumoniae* | 2 (8.0%) | 4 (11.1%) | 6 (9.8%) |
| *Streptococcus pyogenes* | 0 | 2 (5.6%) | 2 (3.3%) |
| Unspeciated gram negative bacteria (rods and diplococci) | 0 | 3 (8.3%) | 3 (4.9%) |
| Unspeciated gram positive cocci in pairs | 3 (12.0%) | 4 (11.1%) | 7 (11.5%) |
| *Candida tropicalis* | 0 | 1 (2.8%) | 1 (1.6%) |
| **Sum Positive Respiratory Culture, *n* (% row total)** | **25 (41%)** | **36 (59%)** | **61 (100%)** |

^a^The following viruses and bacterium are reported on the viral respiratory panel, but were not detected in this cohort of patients: Coronavirus 229E and OC43, Parainfluenza 2 and 4, *Bordetella pertussis*, and *Chlamydia pneumoniae*.

^b^*Mycoplasma pneumoniae* is an atypical bacterium; however, it is detected in the clinical viral respiratory panel and is therefore reported in the tabulation of the viral panel data.

**
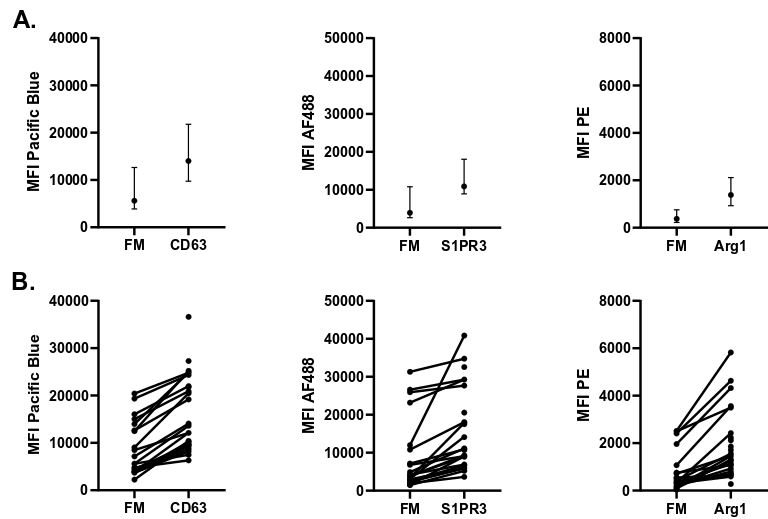
**

**Supplementary Figure S1. Comparison of negative antibody staining controls and patient airway samples.** Median values of mean fluorescent intensity (MFI) for single, live, CD66b^+^ neutrophils for fluorescence minus (FM) controls compared to patient samples. FM controls are only stained with Live/Dead Aqua and CD41a, CD66b, and CD16. **(A)** Median MFIs and 95% confidence intervals for CD63, S1PR3, and arginase 1(Arg1) compared to the FM controls. **(B)** Individual MFIs for each patient’s airway sample is shown with a paired FM control if enough cells were available to perform a FM staining comparison. Using a two-tailed paired t-test, there were 21 sample pairs analyzed for CD63 (*p* < 0.0001) and S1PR3 (*p*  = 0.0002), and 20 sample pairs analyzed for Arg1 (*p* < 0.0001).
